# Supplementary material for: Extracellular matrix signatures of human mammary carcinoma identify novel metastasis promoters
Source: eLife. 2014 Mar 11;3:e01308. doi: 10.7554/eLife.01308 (PMC3944437; doi:10.7554/eLife.01308)
Supplement: Supplementary file 2. — shRNA sequences. DOI: http://dx.doi.org/10.7554/eLife.01308.021 [file elife01308s005.docx]

**Supplementary File S2. shRNA sequences**

The two sets of 21-targeting nucleotides forming the hairpin are underlined.

| **Target Gene Symbol** | **sh#** | **Sequence** |
| --- | --- | --- |
| Control *(Firefly Luciferase)* | sh#Cont. | TGCTGTTGACAGTGAGCG**AGCTCCCGTGAATTGGAATCC**TAGTGAAGCCACAGATGTA**GGATTCCAATTCAGCGGGAGC***C*TGCCTACTGCCTCGGA |
| CYR61 | sh#1 | TGCTGTTGACAGTGAGCG*C***TCCAAGAACGTCATGATGATC**TAGTGAAGCCACAGATGTA**GATCATCATGACGTTCTTGGA***A*TGCCTACTGCCTCGGA |
|  | sh#2 | TGCTGTTGACAGTGAGCG*C***CTACAGGCTGTTCAATGACAT**TAGTGAAGCCACAGATGTA**ATGTCATTGAACAGCCTGTAG***A*TGCCTACTGCCTCGGA |
| EGLN1 | sh#1 | TGCTGTTGACAGTGAGCG*A***CCAAGGTAAGTGGAGGTATAC**TAGTGAAGCCACAGATGTA**GTATACCTCCACTTACCTTGG***C*TGCCTACTGCCTCGGA |
|  | sh#2 | TGCTGTTGACAGTGAGCG*C***ACGCAATAACTGTTTGGTATT**TAGTGAAGCCACAGATGTA**AATACCAAACAGTTATTGCGT***A*TGCCTACTGCCTCGGA |
| LTBP3 | sh#1 | TGCTGTTGACAGTGAGCG*C***TGACACAGTGTTCTGCGACAG**TAGTGAAGCCACAGATGTA**CTGTCGCAGAACACTGTGTCA***T*TGCCTACTGCCTCGGA |
|  | sh#2 | TGCTGTTGACAGTGAGCG*A***GCCGGTGATCTGCAAGCGGAC**TAGTGAAGCCACAGATGTA**GTCCGCTTGCAGATCACCGGC***G*TGCCTACTGCCTCGGA |
| S100A2 | sh#1 | TGCTGTTGACAGTGAGCGC**ATGAAGGAACTTCTGCACAAG**TAGTGAAGCCACAGATGTA**CTTGTGCAGAAGTTCCTTCAT***T*TGCCTACTGCCTCGGA |
|  | sh#2 | TGCTGTTGACAGTGAGCG*A***AGCCTGGATGAGAACAGTGAC**TAGTGAAGCCACAGATGTA**GTCACTGTTCTCATCCAGGCT***G*TGCCTACTGCCTCGGA |
| SNED1 | sh#1 | TGCTGTTGACAGTGAGCG*A***GGCTTCTCTGTGAATTTGAAA**TAGTGAAGCCACAGATGTA**TTTCAAATTCACAGAGAAGCC***C*TGCCTACTGCCTCGGA |
|  | sh#2 | TGCTGTTGACAGTGAGCG*C***CGGGATCATCTCCTTCCTGAA**TAGTGAAGCCACAGATGTA**TTCAGGAAGGAGATGATCCCG***T*TGCCTACTGCCTCGGA |
