# Supplementary material for: Extracellular matrix signatures of human mammary carcinoma identify novel metastasis promoters
Source: eLife. 2014 Mar 11;3:e01308. doi: 10.7554/eLife.01308 (PMC3944437; doi:10.7554/eLife.01308)
Supplement: Supplementary file 3. — qPCR primer sequences. DOI: http://dx.doi.org/10.7554/eLife.01308.022 [file elife01308s006.docx]

**Supplementary File S3. List of qPCR primers**

Species-specific qPCR primers were designed using Integrated DNA Technology’s software (<http://www.idtdna.com/scitools/Applications/RealTimePCR/>).

| **Target Gene** | **qPCR Primer Sequence** |
| --- | --- |
| **Primers specific of human gene sequence** | |
| ACTIN | Fwd : 5’ ACCTTCTACAATGAGCTGCG 3’  Rev : 5’ CCTGGATAGCAACGTACATGG 3’ |
| ANGPTL4 | Fwd : 5’ AGACACAACTCAAGGCTCAG 3’  Rev : 5’ CTCATGGTCTAGGTGCTTGTG 3’ |
| CYR61 | Fwd : 5’ CAAGGAGCTGGGATTCGATG 3’  Rev : 5’ AAAGGGTTGTATAGGATGCGAG 3’ |
| EGLN1 | Fwd : 5’ GACCTGATACGCCACTGTAAC 3’  Rev : 5’ CCGGATAACAAGCAACCATG 3’ |
| LTBP3 | Fwd : 5’ CACTGCGAAATCTACCCCTG 3’  Rev : 5’ GTGTAGCCCTTTCCGTCTG 3’ |
| LOXL2 | Fwd : 5’ GTGCAGCGACAAAAGGATTC 3’  Rev : 5’ GCGGTAGGTTGAGAGGATG 3’ |
| S100A2 | Fwd : 5’ GCCCACATATAAATCCTCACCC 3’  Rev : 5’ GGAGTACTTGTGGAAGGTAGTG 3’ |
| SNED1 | Fwd : 5’ CTACCGAGTTCACCAAGACATC 3’  Rev : 5’ GGTGGAGTGTAACAAGAACGTC 3’ |
| **Primers specific of murine gene sequence** | |
| Actin | Fwd : 5’ TGTATGAAGGCTTTGGTCTCC 3’  Rev : 5’ GTCTCAAGTCAGTGTACAGGC 3’ |
| Cyr61 | Fwd : 5’ GGAGGTGGAGTTAACGAGAAAC 3’  Rev : 5’ GTGGTCTGAACGATGCATTTC 3’ |
| Egln1 | Fwd : 5’ GGTATTTTGATGCAGATGAGCG 3’  Rev : 5’ TGTAGGTGACGTGGGTACTG 3’ |
| Ltbp3 | Fwd : 5’ GCGAAATCTATCCCTGTCCAG 3’  Rev : 5’ CTCTGCCCCAAACAATATGC 3’ |
| S100a2 | Fwd : 5’ CTATACCTTCCACAAGTACGCC 3’  Rev : 5’ TCCTTCTTCACCTTCTCATCATC 3’ |
| Sned1 | Fwd : 5’ GTAGATGGAAGAGGAAGAGTGAG 3’  Rev : 5’ CTGTTCTTGGGTAGCTGGAG 3’ |
